# A Descriptive Analysis of GP Referrals to a Model 4 Emergency Department in the West of Ireland

**DOI:** 10.1007/s11845-026-04282-6

**Published:** 2026-03-03

**Authors:** Marguerite Fortin, Daniel Scott, Safi Ullah, Nur Farah Binti Hussain, Siobhan McGrath, Declan Larkin, Deirdre Kelly, James Binchy, Enda Hession, James Foley

**Affiliations:** 1 School of Medicine, University of Galway, Galway, Ireland; 2 Emergency Department, University Hospital Galway, Galway, Ireland; 3 Headford Road Medical Clinic, Galway, Ireland

**Keywords:** Communication, Emergency Department, General Practitioner, Healthlink, Ireland, Referral

## Abstract

**Background:**

General Practitioner (GP) referrals to Emergency Departments (EDs) are common in Ireland, yet the structure and content of referral letters in Ireland are poorly described. Understanding these patterns may highlight opportunities to optimise communication and streamline patient flow.

**Aim:**

The aim of the study was to describe the characteristics, timing, reasons, and content of GP referral letters to a model 4 emergency department, and to identify areas for improvement for GP to ED communication.

**Methods:**

A retrospective observational study was conducted at University Hospital Galway, a Model 4 ED, analysing all electronic GP referral letters submitted via Healthlink during a two-week period in October 2024. Letters were anonymised, and data on demographics, referral content, and specific requests were extracted. Descriptive statistics and chi-square analysis were performed using SPSS v29.

**Results:**

Of 654 GP referrals reviewed, 644 were eligible for analysis. Most referrals originated within 5 km of the hospital (42%) and presented during working hours (84%). A likely diagnosis was present in 36%, a specific request in 43%, and vital signs in 60% of letters. Imaging (34%) and specialty review (31%) were the most frequent requests. The most common referral themes were injuries (13%), abdominal pain (12%), and infections (8%).

**Conclusion:**

Many GP referrals to this model 4 ED requested imaging or request for a review by a specialty. Documentation within letters was varied. Engaging GPs and highlighting key clinical details could improve documentation and, in turn, improve communication and patient disposition.

**Supplementary Information:**

The online version contains supplementary material available at 10.1007/s11845-026-04282-6.

## Introduction

Healthcare systems across Ireland and internationally are facing increasing demand, with rising attendance rates across outpatient, inpatient, and emergency services [1, 2]. In Ireland, the population has risen by 70% between 1971 and 2022 but healthcare resources have not matched this rise in population [3]. Internationally, it has been shown that General Practitioners (GPs) are increasingly referring patients to emergency departments (ED) for investigations or specialist review that may not be readily accessible in the community, and often there are no alternatives other than the ED, due to waiting lists, lack of community radiological resources, and access to specialty input [4]. Although referrals to ED represent less than 3% of total GP workload, some studies have shown that they can account for a significant proportion of presentations to ED, with one study reporting that GP referrals account for 37% of all ED attendances in Ireland between 2014–2017 [5–7]. Their contribution to overall ED activity highlights the importance of understanding referral patterns to ensure efficient triage and resource allocation.

Previous research in Ireland has explored various factors influencing GP referral patterns, including GP characteristics such as gender and GMS eligibility, policy changes like the introduction of free GP care and barriers such as limited access to diagnostics [5, 8, 9]. Others have assessed the perceived appropriateness of referrals, with Cummins et al. showing GPs and EM Clinicians agreeing that “inappropriate” ED presentations are a problem in Ireland, but there were differences in opinions in what GPs and EM clinicians considered “inappropriate” referrals. [10]. While these studies offer valuable insights into referral drivers, few have examined the actual structure and clinical content of GP referral letters, leaving a gap in our understanding of the handover process between primary and emergency care.

This study aims to provide a comprehensive descriptive analysis of GP referral letters to a model 4 Emergency Department in the Republic of Ireland, focusing on patient characteristics, reasons for referral, and the structure and content of the letters themselves. By examining real-world documentation across a large sample, this research seeks to offer insights into current referral practices, identify areas for optimisation, and contribute to a more informed, collaborative approach to managing patient transition between primary and emergency care.

## Methods

This was a retrospective observational study conducted at the ED of University Hospital Galway (UHG), a model 4 referral centre in the West of Ireland. This ED has an annual census of 80,000 patients, cares for both adults and children, and in 2024, 41% of attendances were referrals from GPs. Consecutive referrals submitted via the national electronic referral system, Healthlink, for a two-week period in October 2024 were reviewed. This period was chosen out of convenience at the time of study conceptualisation and was felt to represent a sufficiently large sample size for the study question.

Referrals were included if they were submitted electronically via the Healthlink system using the standardised GP referral template. Approximately, 70% of GP referrals are via Healthlink. Referrals were excluded if they were handwritten, provided on practice-headed paper, or not submitted through Healthlink. Healthlink is a secure, internet-based messaging service that enables the exchange of patient clinical information between hospitals and medical practitioners, in accordance with the Health Information and Quality Authority (HIQA) National GP Referral Standards [11, 12]. The referral template includes structured fields for patient demographics, clinical history, examination findings, medications, allergies, investigations and the reason for referral [13].

All referrals were anonymised by the study team prior to analysis to remove personally identifiable information (PII) of the patients and their GP*.* The GP practice addresses were recorded prior to anonymisation and converted to distance in kilometres via road from UHG ED. Data were extracted from each referral letter, including patient characteristics and referral characteristics. If attached to the referral, the presence of laboratory and imaging reports was noted, as were any specific requests from the GP regarding investigations, specialist review or hospital admission. Referral themes of the letters were also identified by MF and JF, with no inter-rater reliability performed.

Descriptive statistics were used to summarise the referral characteristics. Thematic categorisation was applied to identify common reasons for referral and recurring clinical themes. Categorical variables (patient characteristics and letter content) were then compared using chi-square analysis on SPSS v.29.

## Results

**Fig. 1:**
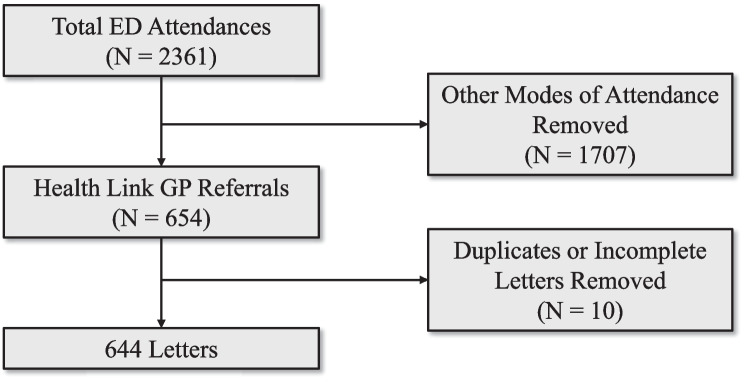
Flow Chart of Letters Inclusion and Exclusion

A final sample of 644 Healthlink letters were included in this study. The flow diagram of the inclusion criteria can be found in Fig. 1.

The characteristics of the patients referred, along with the timing of referrals by day and time, are summarised in Table 1.

**Table 1:** Characteristics of GP Referral Letters to the ED (N = 644)

Patient Characteristics	
Gender	56.2% Female (n = 362)
Age	Median 44 years (IQR 47.0; Range 0.1–95 years)
Age Groupings	Paediatric (< 16 Years): 19.1% (n = 123) Adults: 17–64 Years: 54.0% (n = 347) Older Persons (> 65 Years): 26.9% (n = 173)
Day of Referral	Monday: 21% (n = 136) Tuesday: 21% (n = 134) Wednesday: 19% (n = 119) Thursday: 18% (n = 117) Friday: 21% (n = 134) Saturday/Sunday: 0.62% (n = 4)
Time Period of GP Referral	0900–1200: 31% (n = 199) 1200–1700: 53% (n = 343) 1700–0859: 16% (n = 102)
Distance GP practice to ED	< 5 km: 42% (n = 272) 5-10 km: 5% (n = 34) 11-20 km: 12% (n = 77) 21-30 km: 6% (n = 38) 31-40 km: 11% (n = 68) 41-50 km: 14% (n = 93) > 50 km: 10% (n = 62)

As shown in Table 2, the recommended standards for Healthlink referrals by GPs were analysed. Of the letters included, 64% (n = 409) did not have a likely diagnosis documented, and 57% (n = 369) did not have a specific request for the ED clinician. Vital signs were documented in 60% (n = 388) of referrals.

**Table 2:** Percentage of GP Letters Content (N = 644)

Component of Standard	**Percent (n) in Letter**
Patient Characteristics	100% (n = 644)
Reason for Referral	100% (n = 644)
Likely Diagnosis	36% (n = 235)
Specific Request from GP	43% (n = 275)
Vital Signs	60% (n = 388)
*Laboratory Results	9% (n = 59)
*Imaging Results	3% (n = 22)

^*^Laboratory or imaging results were not relevant for all referrals

**Fig. 2:**
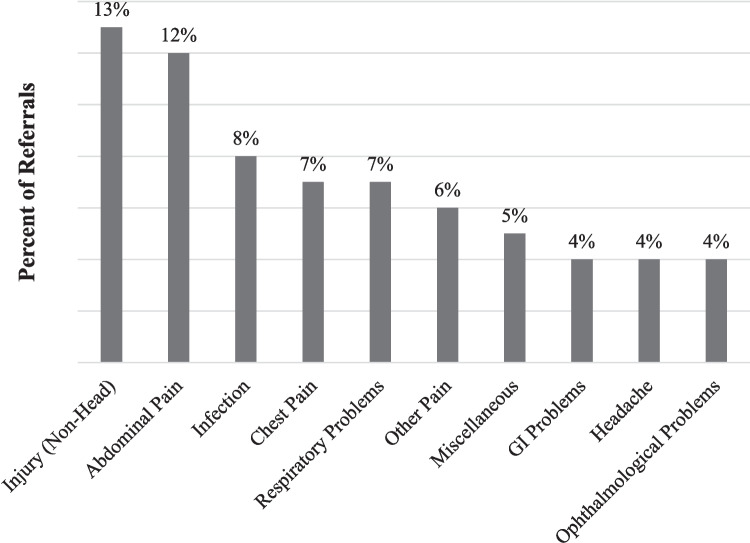
Reasons for Referral

There were 98 referral themes identified for the letters analysed, as shown in the *Supplemental Material*. These were then combined into 25 themes, of which the 10 most common are found in Fig. 2, with the three most common reasons for referral encompassing injuries (excluding head) (13%, n = 86), abdominal pain (12%, n = 78), and infections (8%, n = 52). Of the letters that had a specific request documented (42.7%, n = 275), the most common requests were for imaging (34%, n = 94) which resulted in 125 imaging requests [(plain radiographs (59%, n = 74), computed tomography (20%, n = 25), and ultrasound (6%, n = 8)], followed by review by specialty team (31%, n = 86).

## Discussion

In this two-week analysis of 644 Healthlink GP referrals to a model 4 ED, referrals clustered within 5 km of the hospital and peaked during afternoon working hours, on weekdays. The most frequent reasons were injuries (excluding head), abdominal pain, and infections. It was identified that key elements of letters were frequently missing (e.g., working diagnosis, explicit request, vital signs), while documented requests most often sought imaging or specialty input. GPs undergo a large number of consultations every year, yet they refer less than 5% of these patients to the ED [5, 6]. However, as previous literature has described, this has the potential to translate into a significant percentage of ED attendances [7]. Delays as a result are inevitable, with crowding and congestion being potential consequences, the causes of which are multifactorial and not exclusively caused by patients referred by their GP to EDs [4, 14].

It is reported that EDs and GPs are busier on Mondays [15–18]. Our analysis found that GP referrals to the ED were the same across all days of the working week. Most referrals were sent in the afternoon, which aligns with the typical GP workflow given practice opening hours, and travel time for patients post their GP visit [19]*.* Our study found that a greater number of referrals were made from GP Surgeries located within a 5 km radius of this ED, which contrasts with existing literature by Gouda et al. in County Sligo who reported that referrals increase proportionally with the distance from ED [5]. This pattern may have been observed due to proximity to the hospital, or more likely it is related to higher population numbers or GP practices in Galway city itself. The Central Statistics Office showed 44% of the Galway City population and 4.2% of Galway County population lived within a 5 km radius of an adult ED [20]. However, given referrals also came from counties outside Galway, this likely reflects the tertiary services provided in this hospital, as it is the Model 4 hospital for the HSE West Region.

A study by the Irish College of General Practitioners reported that GPs with access to radiology services were less likely to refer patients to EDs or acute medical units [9]. Our results are in agreement with this, but further explorative research is needed to establish the barriers and enablers to community-based investigations. Specialty requests are likely to be for patients with a deterioration in a chronic illness, or with a new diagnosis for which the GP feels a specialty assessment is required. In this hospital, the HSE tracker reports that outpatient appointments can take an average of 210 days for a first appointment [21]. This may lead to patients being referred to the ED, despite the GP recognising that they need a particular specialist or outpatient review.

Regarding letter content, even though every referral contained patient demographics and the reason for referral (i.e. presenting complaint), most letters lacked some of the additional clinically relevant information. Indeed, over half of the referral letters did not include a likely diagnosis or a specific request from the GP, and less than two-thirds contained vital signs, although not all presentations require vital signs to assist with onward disposition. It could be assumed that the symptoms and signs documented portray the likely diagnosis, but adequate and consistent documentation is likely to improve efficiency and accuracy of triage once the patient arrives to ED. This could improve patient flow throughout their ED visit as it will be clear on the referral letter what the likely diagnosis is and what the suggested management is. This could expedite the clinical journey from the front door, rather than a letter without sufficient information that does not add value to the patient’s care.

The reasons for a lack of documentation are not described in this study, but could centre on administrative burden, and busy GPs who likely do not have sufficient time to review a patient, recognise they need onward disposition to the ED, and generate a letter to match that need in a timely fashion before the next patient knocks on the door. Additionally, and while not analysed in this study, communication from hospitals and EDs to GPs is often inadequate with GPs reporting mixed satisfaction from GP letters received, and other studies reporting no letter received at all from an ED attendance [22–24]. In general, effective communication between GPs and EDs is vital for patient safety and continuity of care, and should be prioritised by both hospital and community-based healthcare providers [11, 25, 26].

The limitations of this study include its retrospective design and the single-centre nature of the data, which reduces the generalisability of the results. Furthermore, not all GP letters were included, resulting in selection bias. Choosing to include Healthlink referrals excluded entire GP surgeries from analysis as some practices use personalised headed paper for referrals, or more rarely, handwritten letters. This excluded a number of patients and explains why the study cohort represent less than 30% of the patients for the study period. Also, as this study only spanned a two-week period in October, the data do not reflect potential seasonal variations in reasons for GP referrals to ED. Thematic conclusions were chosen by the lead author and verified by the principal investigator. Inter-rater analysis was not performed.

This is the first detailed analysis of GP referral letters to an Irish model 4 ED and has implications for national policy and further re-emphasises the use and benefit of the national referral letter. Substantial variation was found in letter content, with many omitting key information. These gaps may hinder triage accuracy, delay diagnosis, and impact patient flow. The reasons for incomplete letters are likely multifactorial, and will vary depending on the patient, pathology and practice. Further thematic exploratory analysis could help to establish an agreement between primary and secondary care regarding the gold standard referral and discharge letter, and the relevant information that is useful for each party. Collaboration is needed between EDs and Primary Care to enhance communication both to and from the ED regarding letter content, and the downstream impacts of a good referral letter on patient flow. EDs should focus on ensuring adequate discharge summaries to ensure GPs are kept informed regarding their patient’s ED visit. Future work should assess the ED outcomes of these patients and if enhanced community access assists GPs caring for patients using alternative pathways.

## Supplementary Information

Below is the link to the electronic supplementary material.

Supplementary file1 (DOCX 19 KB)

## Data Availability

Due to institutional and ethical restrictions, the data underlying this study are not publicly available.
