# Supplementary material for: A Descriptive Analysis of GP Referrals to a Model 4 Emergency Department in the West of Ireland
Source: Ir J Med Sci. 2026 Mar 3;195(3):1681–6. doi: 10.1007/s11845-026-04282-6 (PMC13342269; doi:10.1007/s11845-026-04282-6)
Supplement: Supplementary file 1 — Supplementary file1 (DOCX 19 KB) [file 11845_2026_4282_MOESM1_ESM.docx]

Supplemental Material

| Reason for Referral | Percentage | Reason for Referral (2) | Percentage |
| --- | --- | --- | --- |
| Abdominal Pain | 11.98% (n=77) | Epistaxis | 0.47% (n=3) |
| Chest Pain | 6.84% (n=44) | Hip Injury | 0.47% (n=3) |
| Shortness of Breath | 5.6% (n=37) | Stroke | 0.47% (n=3) |
| Eye Casualty | 4.04% (n=26) | Confirmed Fracture | 0.47% (n=3) |
| Headache | 4.04% (n=26) | Constipation | 0.31% (n=2) |
| Query DVT | 3.27% (n=21) | Diarrhoea | 0.31% (n=2) |
| Back Pain | 2.95% (n=19) | Foreign Body in Ear | 0.31% (n=2) |
| Ankle Injury | 2.95% (n=19) | Hip Pain | 0.31% (n=2) |
| Hand Injury | 2.95% (n=19) | Arm Injury | 0.31% (n=2) |
| Vomiting | 2.95% (n=19) | Coccyx Injury | 0.31% (n=2) |
| Skin Infection | 2.80% (n=18) | Elbow Injury | 0.31% (n=2) |
| Head Injury | 2.49% (n=16) | Hand and Wrist Injury | 0.31% (n=2) |
| Psychiatric Issue | 2.49% (n=16) | Non-Traumatic Leg Pain | 0.31% (n=2) |
| Miscellaneous | 2.18% (n=14) | Limb Infection | 0.31% (n=2) |
| Fever | 2.02% (n=13) | Query Fracture | 0.31% (n=2) |
| Off Baseline | 1.87% (n=12) | Rectal Bleeding | 0.31% (n=2) |
| Abscess | 1.71% (n=11) | Weight Loss | 0.31% (n=2) |
| Foot Injury | 1.56% (n=10) | Burn | 0.16% (n=1) |
| Knee Injury | 1.56% (n=10) | Facial Pain | 0.16% (n=1) |
| Laceration | 1.40% (n=9) | Foreign Body in Nose | 0.16% (n=1) |
| Atrial Fibrillation | 1.24% (n=8) | Generalised Pain | 0.16% (n=1) |
| Obstetric Complications | 1.24% (n=8) | Gynae Foreign Body | 0.16% (n=1) |
| Testicles/Scrotal Problems | 1.24% (n=8) | Haematemesis | 0.16% (n=1) |
| Dizziness/Vertigo | 1.09% (n=7) | Hearing loss | 0.16% (n=1) |
| Knee Pain | 1.09% (n=7) | Arm and Shoulder Injury | 0.16% (n=1) |
| Non-Traumatic Bone Pain | 1.09% (n=7) | Arm Pain | 0.16% (n=1) |
| Rash | 1.09% (n=7) | Back Injury | 0.16% (n=1) |
| Tonsilitis/Quinsy | 1.09% (n=7) | Clavicle Injury | 0.16% (n=1) |
| Vaginal Bleeding | 1.09% (n=7) | Foot Review | 0.16% (n=1) |
| Wrist Injury | 1.09% (n=7) | Leg Injury | 0.16% (n=1) |
| Abnormal Blood Results | 0.93% (n=6) | Lump | 0.16% (n=1) |
| Face Injury | 0.93% (n=6) | Shoulder Injury | 0.16% (n=1) |
| Ear Infection | 0.78% (n=5) | Sternum Injury | 0.16% (n=1) |
| Failure to Thrive (Child) | 0.78% (n=5) | Neck Pain | 0.16% (n=1) |
| Foreign Body in Finger | 0.78% (n=5) | Neonatal Jaundice | 0.16% (n=1) |
| Other Urology Problem | 0.78% (n=5) | Non-Accidental Injury | 0.16% (n=1) |
| Palpitations | 0.78% (n=5) | Other Infection | 0.16% (n=1) |
| Post-Operative Complication | 0.78% (n=5) | Pilonidal Cyst | 0.16% (n=1) |
| Post-Operative Infection | 0.78% (n=5) | Pressure Sore | 0.16% (n=1) |
| Syncope | 0.78% (n=5) | Transient Ischaemic Accident | 0.16% (n=1) |
| Urinary Tract Infection | 0.78% (n=5) | Tinnitus | 0.16% (n=1) |
| Fall (Elderly) | 0.62% (n=4) | Tongue Bleed | 0.16% (n=1) |
| Haematuria | 0.62% (n=4) | Toxicology | 0.16% (n=1) |
| Limp | 0.62% (n=4) | Ulceration | 0.16% (n=1) |
| Seizures | 0.62% (n=4) | Unwell Adult | 0.16% (n=1) |
| Confirmed Fracture | 0.47% (n=3) | Urinary Symptoms | 0.16% (n=1) |
| Dysphagia | 0.47% (n=3) | Varicose Vein | 0.16% (n=1) |
| ECG Findings | 0.47% (n=3) | Vascular Problem | 0.16% (n=1) |
